# Investigation of Calcium Phosphate-Based Biopolymer Composite Scaffolds for Bone Tissue Engineering

**DOI:** 10.3390/ijms252413716

**Published:** 2024-12-22

**Authors:** Monika Furko, Zsolt E. Horváth, Istvan Tolnai, Katalin Balázsi, Csaba Balázsi

**Affiliations:** Institute of Technical Physics and Materials Science, HUN-REN Centre for Energy Research, Konkoly-Thege str. 29-33, H-1121 Budapest, Hungary; horvath.zsolt.endre@ek.hun-ren.hu (Z.E.H.); tolnai.istvan@ek.hun-ren.hu (I.T.); balazsi.katalin@ek.hun-ren.hu (K.B.)

**Keywords:** calcium phosphates, bioactive elements, biopolymers, morphology, biodegradability

## Abstract

We present a novel method for preparing bioactive and biomineralized calcium phosphate (mCP)-loaded biopolymer composite scaffolds with a porous structure. Two types of polymers were investigated as matrices: one natural, cellulose acetate (CA), and one synthetic, polycaprolactone (PCL). Biomineralized calcium phosphate particles were synthesized via wet chemical precipitation, followed by the addition of organic biominerals, such as magnesium gluconate and zinc gluconate, to enhance the bioactivity of the pure CP phase. We compared the morphological and chemical characteristics of the two types of composites and assessed the effect of biomineralization on the particle structure of pure CP. The precipitated CP primarily consisted of nanocrystalline apatite, and the addition of organic trace elements significantly influenced the morphology by reducing particle size. FE-SEM elemental mapping confirmed the successful incorporation of mCP particles into both CA and PCL polymer matrices. Short-term immersion tests revealed that the decomposition rate of both composites is slow, with moderate and gradual ionic dissolution observed via ICP-OES measurements. The weight loss of the PCL-based composite during immersion was minimal, decreasing by only 0.5%, while the CA-based composite initially exhibited a slight weight increase before gradually decreasing over time.

## 1. Introduction

As of today, there are intensive and competitive efforts from different research groups all over the world to develop new types of novel scaffolds in bone tissue engineering, as is extensively discussed and illustrated in some recent review papers [1,2].

It is well known that optimally designed scaffolds can be a promising material to accelerate bone healing, providing the most optimum environment for bone cell attachment and growth inducing new bone formation. This is the reason why enhanced bioactivity is required from the implants. Scaffolding acts as a supportive framework for the cultivation of cells in a controlled environment, with additional cell stimulation promoting the formation of a matrix necessary for constructing a tissue base for transplant purposes. Recent progress in tissue engineering includes the formulation of novel biomaterials designed to suit particular local environments and clinical needs [3,4]. These scaffold materials can be prepared and utilized in various ways, which can determine their mechanical and chemical characteristics, the surface area, porosity, micro-, and nanostructure, thus affecting their biological performance [5,6,7,8,9,10].

One of the best ways to increase the biocompatibility/bioactivity of these biopolymer-based scaffolds is a calcium phosphate phase incorporation as filler material. It is common knowledge that calcium phosphates (in particular the hydroxyapatite phase) are the main mineral constituents of the natural bones and they can greatly assist in bone cell attachment and growth. By utilizing targeted preparation conditions and the correct quantity of biomineral supplements, the chemical properties and biological efficacy of these scaffolds can be effectively tailored to meet the stringent standards necessary for biomedical uses [11,12,13].

The primary idea of applying novel biodegradable biocomposite scaffolds is that they can serve as an intermediary surface that enhances the adhesion, growth, and proliferation of bone cells, along with promoting the formation of new bone tissue. Once the bones are healed, the degradation of scaffolds occurring in the body is considered favorable from both a clinical and biomedical viewpoint. The dissolution rate of the scaffolds can be tailored by embedding the calcium phosphate particles within appropriately selected biopolymer materials. Our thorough investigation and review of the existing literature indicate that cellulose acetate (CA) and polycaprolactone (PCL) are the most appropriate scaffold materials for medium- to long-term applications. A crucial consideration is that the composite scaffolds made from bioceramics and biopolymers break down into harmless by-products, which the body can fully eliminate after the process of ossification [14,15,16]. The PCL is an FDA-approved (FDA: United States Food and Drug Administration) biocompatible synthetic polymer with moderate biodegradability in biological environments [17]. It consists of serial connected hexanoate units and has a polar ester group and five non-polar methylene groups resulting in a slight amphiphilic nature in addition to its intrinsic hydrophobicity [18,19]. Contrarily, cellulose acetate is a natural, biocompatible as well as biodegradable polymer. Practically, it is the acetate ester of cellulose [20]. CA possesses many specific properties that are advantageous in various applications, such as in drug delivery systems, in scaffolds, in medical coatings, in filtration (such as membrane filters), and last but not least, in food packaging [21,22,23,24,25]. Embedding calcium phosphate particles and other bioactive materials into the base polymer matrix greatly affects the composites’ morphology and structure of the composites, thus affecting their biodegradable potential. In our work, amorphous-nanocrystalline calcium phosphate phases were obtained by chemical precipitation from organic calcium and phosphorus sources and a post-treatment method was also used to obtain an optimal biomineralized CP phase that contains the necessary trace elements (Mg, Zn) in an optimized concentration. There are abundant research and review papers detailing the usefulness and benefits of these essential trace elements within the calcium phosphate matrix [26,27,28,29,30,31]. As a novelty, we have thoroughly compared the two types of composites (natural and synthetic-based) and evaluated the morphological changes that the ceramic powder addition caused in the structure of base polymer films, which have not yet been reported in the scientific literature in such detail. We have also performed immersion tests in saline solution to assess its effect on the microstructure and sample mass over time, reflecting their chemical stability.

## 2. Results and Discussion

### 2.1. Morphological Assessment of the Amorphous Apatite, Biomineralized Apatite, and the Two Types of Composites

#### 2.1.1. Scanning Electron Microscope Analysis

The shapes and sizes of the particles within the pure apatite and the biomineralized apatite were compared as well as their incorporation into the cellulose acetate and the PCL matrices studied.

**Figure 1:**
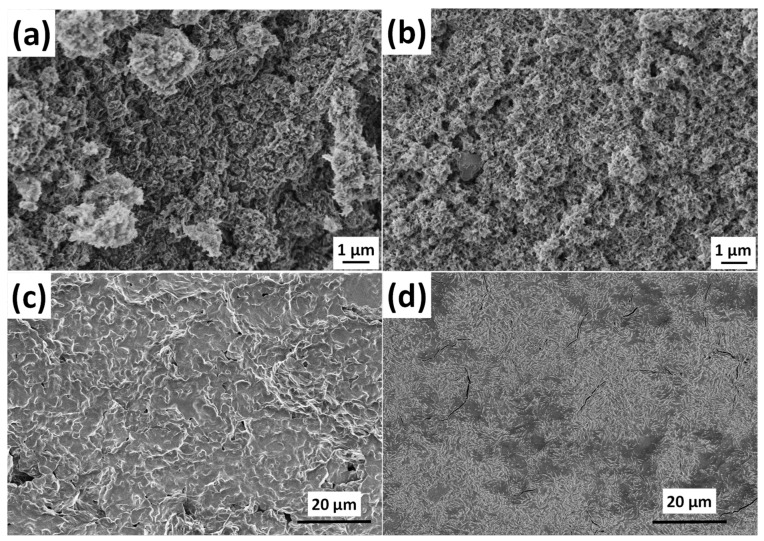

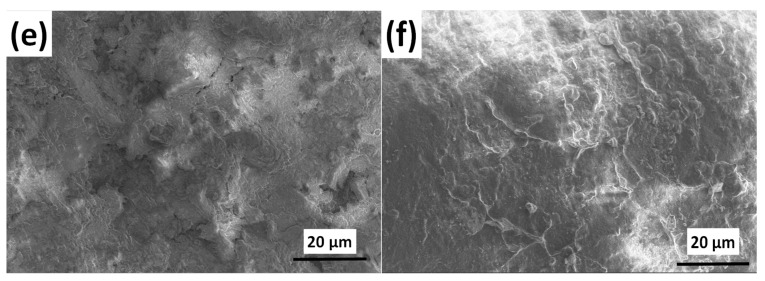
FE-SEM images of amorphous apatite (CP) (**a**) biomineralized (Mg, Zn added apatite (mCP) (**b**), pure cellulose acetate (**c**), pure PCL polymer (**d**), as well as their composites CA-mCP (**e**) and PCL-mCP (**f**). The parameters used in the preparation were kept consistent to ensure their comparability.

As Figure 1 reveals, the CP powder prepared by the wet chemical method from the organic gluconate salt of calcium consists of very small, randomly oriented needle-like particles that form larger, flower-like agglomerations and rounded blocks at some spots. The length of the needle-like particles is around 100–150 nm, but not longer than 200 nm (Figure 1a). On the other hand, the organic Mg and Zn bioactive components added powder has even smaller and densely packed, very thin, thorn-like particles with a seemingly lower number of agglomerates (Figure 1b). This little change in morphology can be attributed to the mineral incorporation into the structure. Moreover, these kinds of needle- or thorn-like structures for various calcium apatites have also been discussed in other research works [32,33,34]. The morphologies of pure cellulose acetate and PCL polymers are shown in Figure 1 c and d, respectively. Both exhibit smooth and homogeneous surfaces. They are formed by mainly shapeless particles and demonstrate a wave-like design. The main difference between the two polymers is that the cellulose acetate film predominantly contains small pores, while the PCL film displays numerous long cracks that disrupt the continuity of the otherwise densely packed particles. In contrast, the composites demonstrate completely different morphologies. The surface of samples contains many small holes and indentations in the cases of both types of polymers. Their structures are predominantly amorphous, with small particles embedded within the base matrix.

**Figure 2:**
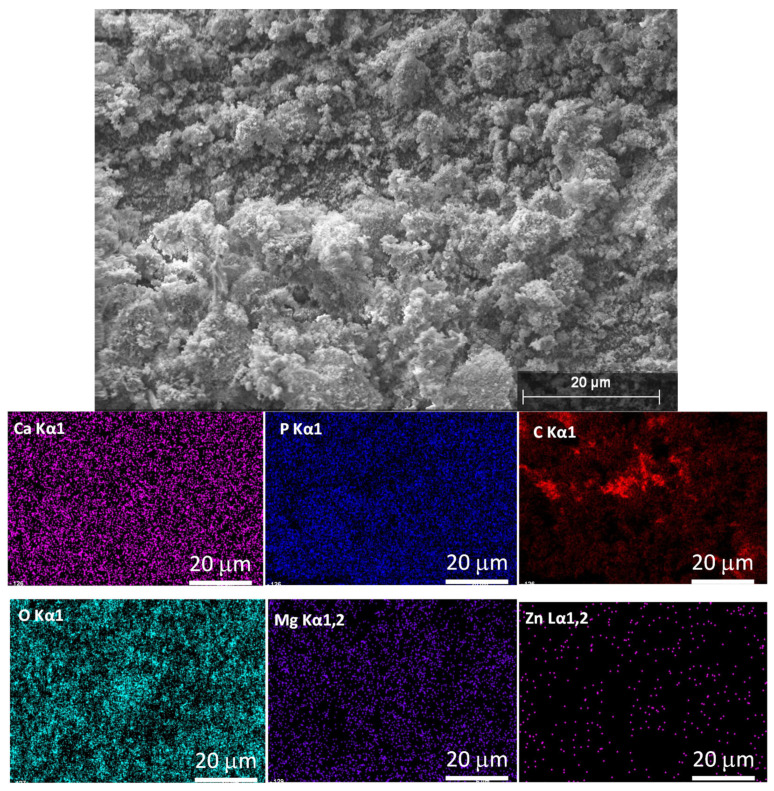
Scanning electron microscope image and the corresponding elemental mapping of biomineralized (Mg, Zn) calcium apatite.

Elemental mapping was performed to check the bioactive elements’ incorporation and distribution in the calcium apatite phase, as shown in Figure 2.

As Figure 2 illustrates, all the doping elements are successfully incorporated into the pure CP matrix. It is also visible that the Mg and Zn element distribution within the investigated area is quite homogeneous and the quantity of the Zn element is lower following the preparation parameters.

**Figure 3:**
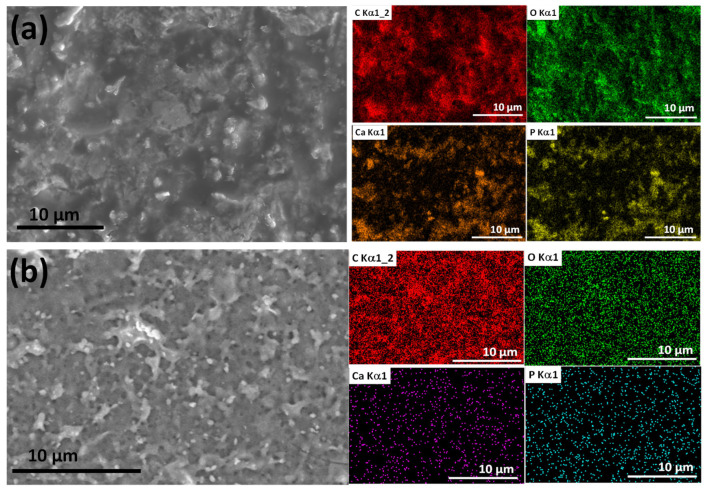
Scanning electron microscope image and the corresponding elemental mapping of PCL-mCP composite (**a**) and CA-mCP composite (**b**).

Additionally, we have also scanned the bioactive mCP powder distribution within the polymer matrices (Figure 3) at lower magnification, covering a larger surface area.

The performed elemental mapping clearly proves the distribution characteristics of the mCP particles. The dispersion of the biomineralized apatite powder is even in both cases; the Ca and P signals are visible in the whole investigated area. However, in the case of PCL polymer, the signals are stronger and denser in some places, which shows their aggregation tendency or the imperfect mixing during the composite preparation. In these samples, the Mg and Zn signals were below the detection limit.

Since the elemental concentration provided by the EDS method is inaccurate, we carried out ICP-OES measurements. The resulting elemental composition and Ca/P ratios of apatite samples can be seen in Table 1.

As can be seen in Table 1, the pure CP sample has a Ca/P elemental ratio of around 1.845, which is higher compared to the value in the hydroxyapatite phase. This can imply that the powder is a specific amorphous calcium apatite that has been reported elsewhere [35,36,37,38]. In the case of mCP, the Ca percentage decreased a little because of the Mg and Zn component addition. The measured percentages reflect sufficiently the elemental ratios applied during the wet chemical precipitation since the Mg and Zn are present in the mCP powder in similar ratios. The calculated Ca/P ratio was around 1.649, which is close to the reported elemental ratio in the hydroxyapatite phase; however, when we take into account the other doping elements, the (Ca+Mg+Zn)/P ratio changes to 2.154. The Mg and Zn elements are reportedly able to incorporate into the calcium phosphate phases, thus making the apatite more similar to the natural bones. It is also described that the Ca to P ratio in human bones can vary between a wide range of around 1.7 and 2.33, depending on the research work and group [39,40]. Comparable broad calcium to phosphorus molar ratios have been reported in the cases of different ion-doped apatites. In a recent research work, Unosson et al. [39] prepared amorphous calcium magnesium fluoride phosphate particles that are usable in preventive dentistry. The particles were prepared by co-precipitation and their amorphous feature originated from substituting Mg^2+^ for Ca^2+^, which impedes the nucleation and growth process of hydroxyapatite crystals. In this case, the Ca/P ratios varied between 1.2 and 2.0, while the Mg content was around 7 Wt.% in the CaP samples. There is another interesting report [40] on the preparation of a Mg- and Zn-added calcium phosphate material where an amorphous calcium phosphate phase co-doped with the Mg and Zn elements was chemically precipitated and transformed into Mg, Zn β-tricalcium phosphate phase by calcination. In this case, the Mg content (in mol%) changed from 5.86 to 8.09, the Zn concentration varied between 0.71 and 2.81, while the calculated (Ca^2+^ + Mg^2+^ + Zn^2+^)/P molar ratio was between 1.47 and 1.56, according to the chemical analyses.

As it is also widely discussed in the scientific literature, the Mg incorporation into the CaP phase can result in a new phase formation, which is the whitlockite (Ca_18_Mg_2_(HPO_4_)_2_(PO_4_)_12_) [41], while the Zn incorporation can form the parascholzite phase (CaZn_2_(PO_4_)_2_⋅2(H_2_O)) [42].

#### 2.1.2. Structural Analyses of CP and mCP Powders and Their Composites with PCL and cA Polymers by XRD Measurements

**Figure 4:**
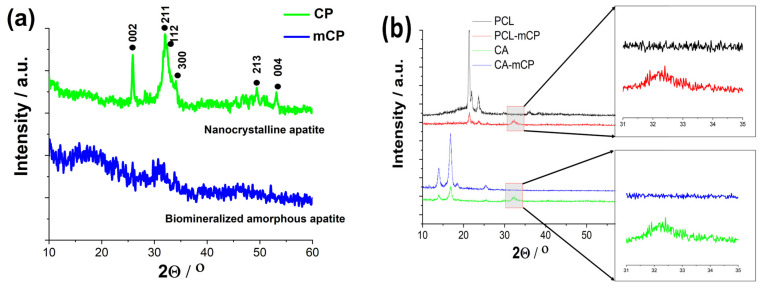
XRD patterns of CP and mCP powders (**a**) prepared by wet chemical method and the PCL-mCP, CA.mCP composites (**b**).

XRD measurements have been performed to determine the phase compositions of CP and biomineralized CP powders and their composites with a naturally derived polymer, such as CA, and a synthetic biopolymer, such as PCL (see Figure 4).

The XRD pattern of the pure CP powder, prepared from organic Ca-gluconate salt, clearly shows the broadened and merged characteristic peaks of nanocrystalline or amorphous apatite with the triple-peak of hydroxyapatite crystal at 2Θ = 31.7°, 32.2° and 32.9° as well as other minor peaks at around 49° and 53°, corresponding to Bragg’s reflection planes 213, and 004, respectively (JCPDS 01-086-1199). This pattern was also reported in other research works [43,44] as a nanocrystalline apatite. It is also visible that the biomineralization process, namely the organic magnesium and zinc component addition to the base CP powder in a relatively low concentration altered the micro- and nanostructure of the resulting powder. The XRD spectrum in this case is a noisy line, with a very small and wide peak emergence at 2Θ from the 30 to 33 regions, which indicates that the powder in this case contains a very small, nano-sized, or even amorphous structure with a random crystal orientation [45,46].

The XRD patterns of the PCL as well as the PCL-mCP composite show the characteristic peaks at 2Θ of 21.3° and 23.6° that correspond to the PCL according to JCPDS file no. 96-720-5590. The pattern of PCL-mCP exhibits noticeably broadened and weak peaks compared to the pattern of pure PCL, and in addition, there are extra merged peaks between 2Θ of 31° and 33° that can be linked to the amorphous apatite (enlarged areas in Figure 4b). This result is well aligned with other works that investigate calcium phosphate-containing PCL composites [47]. For example, Garcia et al. [48] prepared polycaprolactone/calcium phosphates hybrid scaffolds that contained plant extracts with a 3D printing method and proved the incorporation of the CP particles into the polymer matrix. They observed homogeneous distribution of calcium and phosphorus within the filaments. On the other hand, Comini et al. [49] produced a novel poly(ε-caprolactone) (PCL)-based calcium phosphate composites containing silver particles that can be used as bone scaffolds. In their case, the XRD patterns of the composite scaffolds distinctly revealed the unique peaks associated with hydroxyapatite (HA) and beta-tricalcium phosphate, in addition to those of PCL.

The pattern of the cellulose acetate sample has the main characteristic peaks at around 14°, 16.7°, 18.5°, and 25.5°, which are the main characteristic peaks of cellulose acetate according to other research works [50]. The CA-mCP composite sample presents a lower degree of crystallinity than the pure CA sample with widened peaks and lower intensity, similar to the PCL-mCP sample. The extra peaks related to the apatite are also visible in the 31°–33° 2 theta region (also in Figure 4b).

#### 2.1.3. Short-Term Immersion Measurements

We have monitored the changes in both morphology and weight of composites over a short-term (two weeks) period of immersion to check the biodegradability capacity of composite samples and to gain insights into the morphological changes caused by the soaking procedure in general. In addition, we measured the concentrations of the dissolved calcium, magnesium, zinc, and phosphorus ions by ICP-OES at different time intervals.

**Figure 5:**
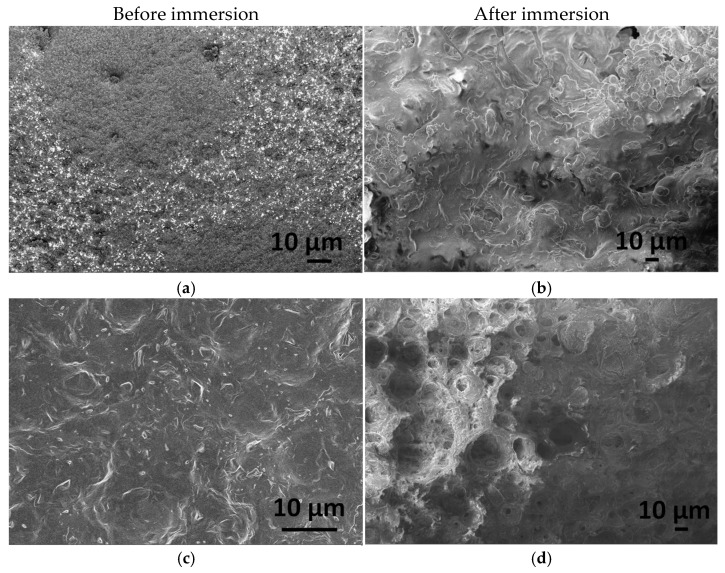
FE-SEM images on CA-mCP (**a**) and PCL-mCP (**c**) composites as prepared as well as CA-mCP (**b**) and PCL-mCP (**d**) composites after two weeks of immersion in saline solution.

Figure 5 illustrates the morphology of CA-mCP and the PCL-mCP samples as prepared and after two weeks of immersion in saline solution.

The differences in morphology before and after immersion in saline are noticeable for both CA- and PCL-based composites. The start of degradation of both polymer composites can be detected by observing how the morphology changes during the immersion period. After immersion, the shapes of the polymer particles became poorly outlined, resulting in a fused shapeless structure that surrounds the mCP granulates. The number of pores increased after soaking, and they became larger. This larger porous structure is more outstanding in the case of the PCL-mCP composite. Generally, both types of polymers’ decomposition happen faster than the CP phase dissolution, since the calcium phosphate materials intrinsically possess a lower degradation rate which means lower solubility [51].

Numerous studies in the scientific literature have explored the dissolution and degradation characteristics of both CA [52,53,54] and PCL polymers [55,56], as well as their composites with bioceramics in biological environments [57,58,59], supporting and reflecting our results.

We also monitored the possible weight changes in samples immersed in the saline solution.

**Figure 6:**
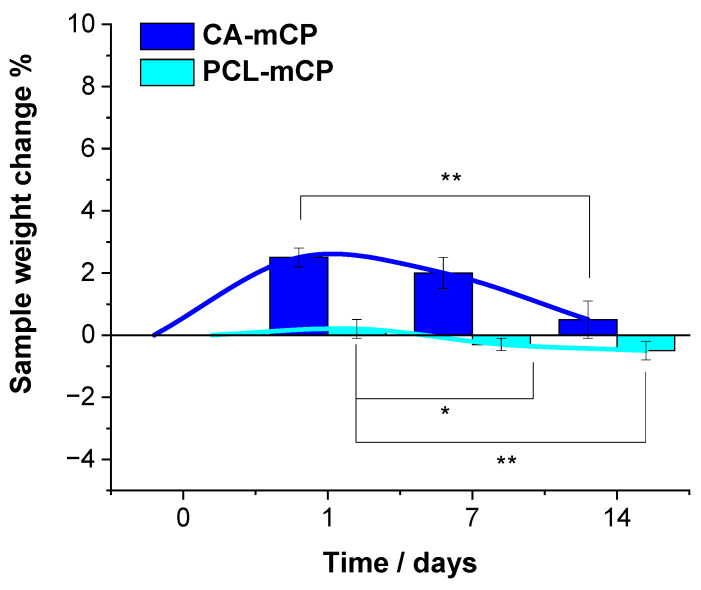
Sample weight changes during the two-week immersion period in saline solution at room temperature. Values are graphed as the mean ± standard deviation (*n* = 3). * indicates *p*  <  0.05; ** indicates *p* <  0.01.

As Figure 6 reveals, the weight change in the PCL-mCP sample is almost negligible, it only decreases by 0.5 percent over the short-time immersion period, remaining almost constant within the margin of error, which proves its moderate degradability. However, the statistical analysis revealed that the difference in weight loss is statistically different after one week of immersion and highly different after two weeks. This characteristic of the PCL polymer was described elsewhere [60], stating that the PCL degradation might occur from a few months to several years depending on its molecular weight, crystallinity rate, porosity, micro-, and nanostructure as well as the polymer thickness, and the environment [61,62]. On the other hand, the CA-based composite showed a very slight increase in weight, but this increasing tendency changed after one week of immersion. The difference in weight change was statistically highly different after two weeks of soaking, but after one week, there was no statistically significant difference between the values. This phenomenon can be explained by the fact that the nature of cellulose acetate is hydrophilic and easily can adsorb and bind water molecules and or mineral salts within its structure even in a dried state. This result is in accordance with the reported findings in the scientific literature [20,63,64,65,66,67,68].

**Figure 7:**
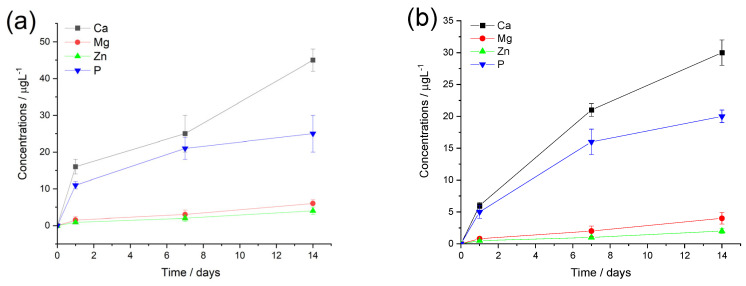
Cumulative concentrations of the dissolved bioactive ions from CA-mCP (**a**) and PCL-mCP (**b**) composites soaked in saline solution at room temperature. The values are normalized to the unit area of samples. All data points are presented as the mean ± standard deviation (*n* = 3).

The ion release curves of both polymer composites show a similar pattern (see in Figure 7), such as the calcium and phosphorus concentrations are higher at each time point which is due to their higher amount in the composite materials. The measured concentrations of Mg and Zn ions are around 10 times lower and show a slight and gradual increasing tendency over time. On the other hand, the phosphorous concentration demonstrates a saturation curve which means a fast increase in values at the early stage of immersion then reaches a saturated state owing to the possible dissolution/precipitation processes when insoluble phosphate precipitate forms. The calcium ion has almost similar curve feature (a semi-saturation curve), which can imply mainly calcium phosphate phase precipitates; however, after around one week of immersion, they start to increase again in the cases of both CA and PCL matrices. This tendency has also been observed in other research works regarding ion-loaded bioceramics [69,70,71]. It is also mentionable that the measured ion concentrations are systematically higher for the CA-based composites owing to the base polymer’s unique chemical characteristics and hydrophilicity.

To date, the main application of these composites is as scaffolds, bone substitutes, or bone grafts. There is intensive research to develop composites with the most suitable chemical and mechanical properties. For instance, Vella et al. [72] prepared calcium phosphate-PCL composite scaffolds using 3D printing to achieve appropriate porosity and degradability to enhance osseointegration. In their work, they applied specific parameters to improve the mechanical strength and toughness of these scaffolds. They employed sintering, and post-print precipitation as well as 3D print co-deposition of PCL with calcium phosphate scaffold matrices. In other more recent work, Garcia et al. [48] developed calcium phosphate nanoparticle-loaded polycaprolactone scaffolds, and then impregnated them with extracts of Colombian plants as antibacterial agents. They concluded that these scaffolds are promising candidates for bone defect filling and regeneration. On the other hand, Shi et al. [73] recently published a review paper on calcium phosphate-containing cellulose-based composites in which they revealed the importance and wide variety of these composites. They stated that these composites are ideal materials as bone scaffolds due to their suitable mechanical properties, biocompatibility and moderate biodegradability.

## 3. Materials and Methods

### 3.1. Preparation of Different Calcium Phosphate Nano-Powders

The previously optimized calcium phosphate phase was prepared by wet precipitation method, dissolving calcium gluconate (C_12_H_22_O_14_Ca, Molar Chemicals Ltd. (Halásztelek, Hungary) —≥99.0%) and disodium hydrogen phosphate (Na_2_HPO_4_, VWR International Ltd.—99%, AnalaR NORMAPUR) as described in our previous paper [30]. The pH of the suspension was adjusted to 11 with 50 g/L sodium hydroxide (NaOH, VWR International Ltd.—≥99.5% ACS, at a pH value of 11) for 24 h to promote the apatite formation. After the apatite formation was completed, bioactive substances were added to the suspension in calculated concentrations. The bioactive materials were organic magnesium gluconate (C_12_H_22_O_14_Mg, Molar Chemicals Ltd.—≥99.0%) and zinc gluconate (C_12_H_22_O_14_Zn, Molar Chemicals Ltd. —≥99.0%) salts that are all extensively used as nutritional supplements to treat magnesium and zinc deficiency in patients. The calculated Ca/Mg/Zn elemental weight ratio was 1/0.2/0.1. Finally, the powders were collected and used for further characterization and composite preparation.

### 3.2. Preparation of mCP-PCL and mCP-CA Composite Scaffolds

For the PCL-based composite preparation, (Polycaprolactone, average M_w_ ~1,300,000, Sigma-Aldrich, St. Louis, MI, USA)) the concentration of the polymer solution was 10% (*w*/*v*) in dichloromethane (DCM) solvent. The composite was formed by adding freshly prepared mCP particles into the polymer solution in 0.2 g/10 mL concentration under vigorous stirring (at 1400 rpm using magnetic stirrer (Heidolph Instruments Gmbh, Schwabach, Germany)).

The same preparation parameters were applied in the case of CA (cellulose acetate average M_w_ ≈ 100,000, Acros Organics, Geel, Antwerp, Belgium, acetyl content 39.8%) biopolymer but the applied solvent was acetone. The composite’s concentration was 0.2 g mCP in a 10 mL polymer solution, similarly. The final suspensions in both cases were poured into silicon molds (in the shape of a disc of 30 mm diameter) and left to dry under ambient conditions. The dried composite films were then collected and further characterized.

### 3.3. Characterization Methods

#### 3.3.1. X-Ray Diffraction Analysis

The structure of biomineralized CaP powder and composites were analyzed with an X-ray diffractometer (XRD, Bruker AXS D8 Discover with Cu Kα radiation source, λ = 0.154 nm) using Göbel mirror and scintillation detector (Bruker AXS, Karlsruhe, Germany). The operation parameters were at 40 kV and 40 mA. The diffraction patterns were collected from the 10° to 65° 2θ range with 0.3°/min steps and 0.02° step size. Diffrac.Eva software 3.1 was used to evaluate the measured XRD patterns and to detect the potential crystallite phases.

#### 3.3.2. Field Emission Scanning Electron Microscopy (FE-SEM)

The morphological properties of calcium phosphate powders, as well as the pure and bioceramic loaded PVP and CA fibers, were examined by field emission scanning electron microscope (FE-SEM, Thermo Scientific, Scios2, Waltham, MA, USA) and Energy Dispersive X-ray Spectrometry (Oxford Instrument EDS detector X-Max^n^, Abingdon, UK). Map sum spectrum was recorded on samples using 6 keV accelerating voltage.

#### 3.3.3. Short-Term Immersion Tests

The bioresorbable characteristics of CA-mCP and PCL-mCP composite samples were studied by a simple immersion test. All the samples were soaked in saline solution (0.9% NaCl solution). The size and shape of the samples was 30 mm × 1 mm discs. The working surface area (in connection with liquid during immersion) of all composites was set to 15 cm^2^. During the soaking procedure, the composite samples were immersed in 10 mL of saline solution in separate containers at room temperature for two weeks. Three samples of each type at each time point were used to ensure parallel measurements and reproducibility. The purpose of this test was to monitor both the quantitative changes in the mass of the composites during soaking and the qualitative changes in the morphology of the composite particles. The percentage of weight change was determined by the following formula: W% = (Ws − Wd)/Ws × 100%; Ws is the starting weight of the sample and Wd represents the weight of the dried samples after different soaking intervals (0, 1, 7, and 14 days). At each specified interval, the samples were removed, thoroughly washed with distilled water, and dried to a constant weight at 50 °C.

#### 3.3.4. ICP-OES Measurements

To determine the exact elemental ratio in CP and mCP powders and to check the possible dissolution of different ions (Ca^2+^, Mg^2+,^ and Zn^2+^) from the composites, an inductively coupled plasma optical emission spectroscopy (ICP-OES) technique with ICP-OES spectrometer (PerkinElmer Avio 200, Waltham, MA, USA) was utilized. The measurement was carried out in a cyclone spray chamber in the presence of an internal standard (1 ppm Y). Four-point calibration was applied, and standard solutions in concentrations of 0.01, 0.1, 1, and 10 ppm were recorded for each element. The CP and mCP powders were dissolved in 10 mL 1 N HCl solution to determine the elemental composition.

To assess the short-term dissolving characteristics of both composite types, samples (with a particular working area of 10 cm^2^) were immersed in 5 mL of saline solution. Samples were collected from the supernatant at different time intervals: 0, 1, 7, and 14 days. The concentrations of Ca^2+^, Mg^2+^, Zn^2+^ ions were measured.

#### 3.3.5. Statistical Analysis

The differences between experimental groups were evaluated by one-way analysis of variance (ANOVA, Origin 2024, Northampton, MA, USA). For the comparison of the mean values, the Tukey test was used. The level of statistical significance was given by a *p*-value of 0.05. A *p* value lower than 0.05 was considered statistically significant (* *p* < 0.05). *p*-values were more highly statistically significant when ** *p* < 0.01. The number of samples per group was N = 3.

## 4. Conclusions

Biomineralized and nanocrystalline CP powders were successfully prepared using the wet chemical method from the organic gluconate salt of calcium. The prepared powder consists of very small, randomly oriented needle-like particles with some agglomerations and larger blocks. The organic Mg and Zn bioactive components addition caused a change in the morphology, the particles became smaller and more densely packed with a lower number of agglomerates.

The XRD measurements also have proven the CP powder to be nanocrystalline or quasi-amorphous, while the mCP powder to be completely amorphous showing only widely broadened and small characteristic peaks. The mCP powder contained the Mg and Zn trace elements in 15 Wt.% in total with a Mg: Zn ratio of around 3:1 as the ICP-AES measurement revealed.

The mCP powder addition to the base polymer solutions (both CA and PCL) also induced a noticeable change in the polymers’ microstructure. The bioceramic particles were evenly distributed into the polymer matrices, according to the FE-SEM elemental mapping.

The short-term immersion tests confirmed the moderate degradability of both types of composites; however, their degradation characteristics were different. This can be attributed to the different physical and chemical properties of the two polymers since the cellulose acetate is hydrophilic whilst the PCL is mainly hydrophobic. Additionally, the ICP measurements detected continuous and gradual ionic dissolution from the composites in both cases.

Owing to the intrinsic biodegradable nature of these polymers, they can be innovatively utilized as resorbable bone fillers or bone grafts in bone tissue engineering or even in drug-incorporating systems for controlled drug release.

## Figures and Tables

**Table 1:** Mean (±SD) elemental percentages in Wt% of the CP powder as well as mCP power (N = 3).

Samples	Ca	P	Mg	Zn	Ca/P	Ca+Mg+Zn/P
CP	65.5 ± 0.98	35.5 ± 0.52	-	-	1.845	-
mCP	52.3 ± 0.87	31.7 ± 0.73	11.9 ± 0.09	4.1 ± 0.03	1.649	2.154

## Data Availability

Not applicable.
